# Poverty modeling in the Islamic Republic of Iran using an ANFIS optimized network with the differential evolution algorithm (ANFIS_DE)

**DOI:** 10.1016/j.mex.2020.101120

**Published:** 2020-10-30

**Authors:** Fateme Nazari Robati, Hossein Akbarifard, Seyyed abdolmajid Jalaee

**Affiliations:** Department of Economics, Faculty of Management and Economics, Shahid Bahonar University of Kerman, Kerman, Iran

**Keywords:** Poverty-MPI-modeling-ANFIS-differential evolution algorithm

## Abstract

Poverty is a multifaceted phenomenon that its study and analysis from all dimensions requires accurate knowledge. In the past, poverty was measured only by the income approach. That is, only people's incomes were compared to the poverty line. But this approach does not identify other dimensions of poverty. Given the importance of discussing poverty in the economies of developing countries, this article examines and models poverty in the Islamic Republic of Iran. This article presents the internal and external dimensions of poverty in the period 1996–2017.

In this paper, to model the poverty in Iran, the ANFIS method optimized with a differential evolution algorithm was used. In this method, a differential evolution algorithm was used to train the ANFIS system instead of the FIS system. To evaluate the strength of the model, mean squared error (MSE), root mean squared error (RMSE), Mean absolute error (MAE), STD_error, Mean_error criteria have been used. The data used in this paper are from the World Development Index (WDI) Database, the World Bank Good Governance Indices, the Heritage Foundation's Economic Freedom Indices, and the United Nations data. This information is related to Iran and in the period (1996–2017). The purpose of this paper is to train the ANFIS network with DE algorithm using time series data and to model the data related to the Iran Multidimensional Poverty Index using the trained network. Multidimensional Poverty Index is a very suitable index for monitoring data. Poverty is in society. With the help of this data, we can assess the trend of poverty and income distribution and welfare in this country. The results of this study showed that training the ANFIS system by differential evolution algorithm, can make a very good improvement in the modeling process and reduce error criteria and improve the accuracy of this method.

• This article has been compiled with the aim of modeling poverty in the Islamic Republic of Iran.
• The method used in this paper is ANFIS network training using the differential evolution algorithm
• The use of evolutionary algorithms to train fuzzy systems and artificial neural networks leads to improved performance.

## Graphical abstract

**Figure:**
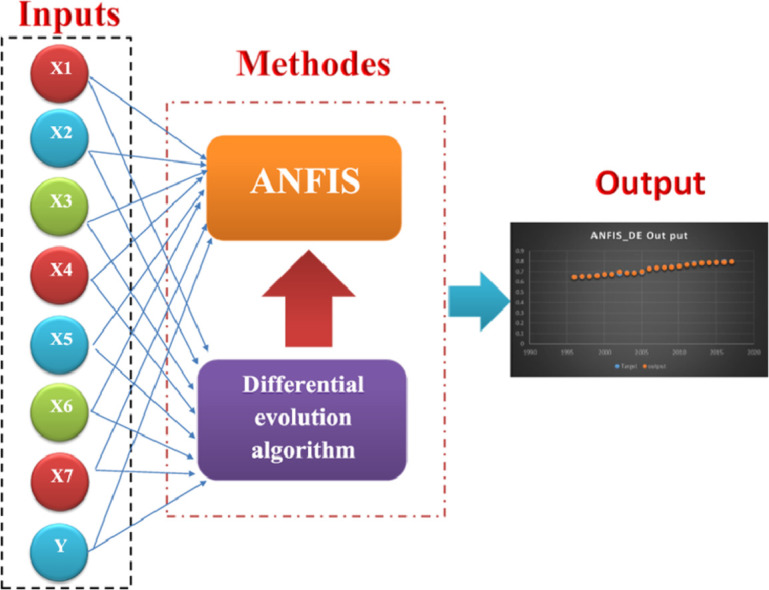

Specifications table

**Table:** 

Subject Area	Economics and Finance
More specific subject area	Macroeconomics - Monetary Economics
Method name	Adaptive Neuro-Fuzzy Inference System (ANFIS) is optimized with the differential evolution algorithm. (ANFIS-DE)
Name and reference of original method	**ANFIS**: Jang J.S.R., Sun C.T., and ME. Neuro-fuzzy and Software Computing: a Computational Approach to Learning and Machine Intelligence. In: Prentice-Hall, New Jersey. 1997. **Differential evolution algorithm:** Storn, R., & Price, K. (1995). Differential evolution–a simple and efficient adaptive scheme for global optimization over continuous spaces: technical report TR-95-012. International Computer Science, Berkeley, California.‏
Resource availability	Data source location: https://info.worldbank.org/governance/wgi/ https://www.heritage.org/index/explore?view=by-region-country-year&u=637188530188497349 https://databank.worldbank.org/reports.aspx?source=world-development-indicators# http://www.hdr.undp.org/en/data# **Software:** MATLAB

## Introduction

Poverty is one of the fundamental problems of human societies and is a clear sign of economic, social, and cultural development. Poverty threatens political stability and social solidarity in society. Poverty reduction as the first goal of the Millennium Development Goals (MDGs) is the biggest global challenge. For this reason, identifying poverty and poverty alleviation methods is one of the most important issues facing policymakers and planners. In recent decades, poverty has been addressed by many international and global organizations. Poverty is a multifaceted phenomenon that requires the study and analysis of all aspects and dimensions. In the past, poverty was measured only by the income approach. But this approach cannot identify all dimensions of poverty. For example, a person may not be poor in income but have to spend a large portion of their income on treatment for illness. That's why the Oxford Institute for Poverty, in collaboration with the United Nations Development Program, presented the Multidimensional Poverty Index (MPI) in 2010. The multidimensional poverty index indicates the deprivation of members of society in basic human capabilities. And presents a different model of income poverty.

The Multidimensional Poverty Index was first calculated by Alkier and Foster in 2007 for 109 countries, and then expanded in 2010 by the Oxford Institute for Poverty and Human Development (OPHI) and the United Nations Development Program. The Multidimensional Poverty Index is calculated annually by the OPHI Institute and the results are published on its official website. The Multidimensional Poverty Index is an international measure of acute poverty, and more than 100 developing countries have calculated it. To calculate this index, various non-income factors have been used to determine poverty. This index complements the measurement of income-based poverty because it includes considerations based on severe deprivation in the dimensions of education, health, and living standards, and in general, its constituent components represent the existence of welfare in the household. The focus of various aspects of development on the monetary and income approach is one of the reasons for the failure of poverty alleviation programs in developing countries. Accordingly, the main cause of poverty is not a lack or lack of income, but the lack of ability of poor people to get out of poverty. The Multidimensional Poverty Index calculates these necessary capabilities in people's lives [1].

The Multidimensional Poverty Index can provide a complete picture of people living in poverty. Also, the calculation method allows comparisons to be made between countries and different regions of the world in terms of poverty, and it is also possible to make an in-country comparison between different ethnic groups, households living in rural or urban areas. These characteristics have made the Multidimensional Poverty Index an analytical tool for identifying vulnerable people. Also, the multidimensional poverty index reveals the pattern of poverty in countries and over time, enabling policymakers to effectively target resources and design more effective policies to alleviate poverty.

One of the major challenges in the field of economic research in today's world is modeling and finding appropriate solutions to reduce poverty in societies. The problem of poverty and income distribution are among the most widely used issues in macroeconomics, and finding appropriate methods for and explaining them is one of the issues of interest in economic discussions. So far, many classical linear models have been proposed for this subject. But the issue of poverty is a complex and non-linear issue that cannot be simulated and analyzed using classical linear methods. Researchers are always looking for more powerful and accurate ways to identify and predict poverty-related parameters by inventing sciences such as intelligent methods. Among these, artificial neural networks and fuzzy neural networks are suitable tools for estimating and predicting many parameters [2].

One of the methods used in recent years to explain phenomena with high complexity is the use of intelligent systems such as artificial neural networks, evolutionary algorithms, and fuzzy logic, which is derived from the human mental structure. The ANFIS inference system can be considered as a combination of neural network and fuzzy systems. Which simultaneously uses neural network learning algorithms and fuzzy logic qualitative expression to design nonlinear mapping between input and output space. This method is widely used today for modeling and simulation in various problems [3]. In neural networks and fuzzy neural networks, determining the appropriate network structure and selecting their parameters is of particular importance. Also, the success of these networks largely depends on the accuracy and efficiency of their learning algorithms. Various algorithms are used to learn neural networks, the most widely used of which are gradient-based algorithms, especially post-diffusion and least squares. Although these algorithms have a lot of capabilities, there are some major weaknesses in them, in some cases create problems for users. , Gradient-based algorithms use, the local search technique and, therefore, are always exposed to the local optimization trap. Algorithms such as Levenberg–Markvrt (LM) also have high computational complexity [4].

Therefore, the use of methods to solve problems of gradient-based algorithms has always been of interest to researchers. One of the most appropriate methods is the use of evolutionary algorithms (EA) to train the network. Evolutionary algorithms have a great ability to perform a global search and avoid local optimization [5,6].

In recent years, optimization algorithms have been used to improve the performance of artificial neural network models in modeling and predicting various problems.

The ANFIS structure consists of several nodes in different layers that are interconnected. The output of this network depends on the parameters that can be adjusted in the nodes. Network learning rules determine how these parameters are updated to minimize errors. The structure of a FIS consists of three main components. Rules database, database, and an argumentation mechanism. A fuzzy rule database contains the IF_THEN rules. The database implements the membership functions used in fuzzy rules as well as the reasoning mechanism, the procedure for inferring the output of input variables. In this article, the DE evolution algorithm is used to teach the ANFIS network. . Due to its simple structure and implementation, fast convergence, and stability, this algorithm has become one of the most widely used evolutionary algorithms to optimize a wide range of problems [7].

According to studies that have been done so far in the field of poverty, several factors affect poverty. A group of economists believes that globalization and trade liberalization pave the way for the transfer of technology and financial resources to developing countries. Multinational corporations reduce production costs, increase employment, and access large export markets by investing in developing countries. In this way, they will increase prosperity and reduce poverty in these countries. In this regard, Saterland believes that in countries where foreign investment is well done and trade liberalization has taken place at a high level, the level of wages has risen significantly. And local standards in technology management and environmental protection have been upgraded [8].

Changes in the general level of prices are another important and influential variable in the level of poverty and instability in the economy, which is usually measured by various indicators such as inflation. Changes in the general price level can affect poverty indicators in two ways. First through revenue effects. That is a change in the real income of households, which is reflected in the reduction of real wages and the devaluation of assets. And second, the change in relative prices, which is interpreted as distributive change. In countries where the government plays an important role in the economy, the term is a kind of flow of wealth from those with fixed incomes to those to whom the increase in liquidity belongs. This leads to the spread of economic problems and the spread of poverty in society. Another variable affecting poverty is the proportion of the rural population in a country. Because with the increase in rural population due to infrastructure and infrastructure in rural areas, economic growth decreases, and the rate of poverty and income inequality increases.

Corruption is the enemy of justice, wealth, and trust, and Anderson outlines several mechanisms by which lawlessness and poverty are linked. Among other things, the poor are more vulnerable to authoritarian behavior [9].

Also, according to the model proposed by Ackerman And Krueger, corruption can affect poverty through economic factors and variables. In other words, increasing corruption leads to reduced investment and helps reduce competition by increasing economic inefficiency. Thus, as competition decreases and trade costs increase, income inequality and poverty in the economy increase [10,11].

Given the above, the model used in this study is by Eq. (1):

(1) Y=f(x1,x2,x3,x4,x5,x6)

wherein Y: Multidimensional Poverty Index (MPI),x1: Rural population, x2: inflation, x3: control of corruption, x4: government effectiveness, x5: property rights, x6: Trade freedom.

In this paper, the Alkire and Foster method is used to calculate the multidimensional poverty index. This method is one of the most widely used multidimensional poverty measurement methods. This method is based on counting used by the United Nations to produce a multidimensional global poverty index. In this paper, MPI is used to model poverty. To achieve the multidimensional poverty index, 10 indicators in three dimensions of education, health, and living facilities were examined. Considering the same weight for each dimension and the same weight for each of the indicators within each dimension, a poor household was identified that was deprived of more than one-third of the indicators. Then, after identifying the poor, the conventional aggregation functions were performed with adjustments. MPI was calculated by Alkire and Foster method. The rural population ratio variable is one of the factors affecting poverty in Iran. These data are calculated and presented by WDI for Iran. In these data, the rural population is reported as a proportion of the country's total population. Another factor affecting poverty is the general level of prices. Consumer inflation data were used for this purpose. This data is obtained from WDI. Data on corruption control and government effectiveness ratings were used to observe the effect of corruption and government on poverty in Iran. These data came from World Bank Good Governance indicators. Also, data related to the observance of property rights and the commercial liberalization index were used to extract MPI data in Iran. These data come from the Heritage Institute's economic freedom indexes [12,13].

To model poverty in Iran according to Eq. (1), the ANFIS method trained by the DE algorithm was used. This system tries to eliminate some of the weaknesses of the default algorithms, such as high computational volume and the possibility of being trapped in local optimization. First, the basic structures of the system are done. Then some parameters of the DE algorithm such as number of iterations, number of the initial population, final optimization limit, coefficients of DE algorithm, the objective function (MSE in this paper), and other parameters related to the algorithm are set. Also, the data is normalized to increase accuracy. The DE algorithm was then selected as the ANFIS trainer and began training the system. his algorithm tries to find the most appropriate model for the MPI based on input variables. After modeling, the accuracy of the presented models is checked with statistical indicators.

## Method details

### Adaptive neuro-fuzzy inference system

One of the methods that in recent years to predict phenomena that affect the very complex factors is the use of intelligent systems such as artificial neural networks, evolutionary algorithms, and fuzzy logic that is derived from the structure. It is the human mind. The adaptive fuzzy-neural inference system (ANFIS) can be introduced as a combination of neural network and fuzzy systems that simultaneously uses neural network learning algorithms and fuzzy logic qualitative expression to design nonlinear mapping between input and output space. It is widely used today in modeling and simulation issues [14].

For the first time, Jang (1993) was able to use the linguistic power of fuzzy systems that conform to the then and then rules and neural networks due to their teaching capabilities and the use of various system training models called fuzzy inference systems based on adaptive neural networks. In this hybrid method, the fuzzy part creates a relationship between the input and output variables, and the parameters of its membership functions are determined by the neural network.

The structure of ANFIS has 5 layers in total. These layers are:

**Layer 1**: This layer is known as the input layer. In this layer, each node i generates membership values that belong to each of the appropriate fuzzy sets, using the membership function defined for it. For example, for input And two membership functions can be written Eqs. (2) and (3):

(2) $$Q_{1,i}=\mu_{A_{i}}\left(x\right)\;for\;i=1,2$$

(3) $$Q_{1,i}=\mu_{B_{i-2}}\left(x\right)\;for\;i=3,4$$

**Layer 2**: In this layer, the input values to each node multiply by the weight of the rules. For the first node, We weight Eq. (4):

(4) $$Q_{2,i}=W_{i}=\mu_{A_{i}}\left(x\right)*\mu_{B_{i}}\left(x\right)\;i=1,2$$

**Layer 3**: Each node in this layer performs the calculation of the relative weight of the rules using the Eq. (5):

(5) $$Q_{3,i}=\bar{W_{i}}=\frac{W_{i}}{\sum_{i=1}^{N}W_{i}}$$

where *N* is the number of nodes in this layer.

**Layer 4**: This layer is known as the rule layer and is obtained from the sum of operations on the input signals to this layer. For this layer we can write Eq. (6):

(6) $$Q_{4,i}=\bar{W_{i}}f_{i}=\bar{W_{i}}\left(p_{i}x+q_{i}y+r_{i}\right)$$

Where $\bar{W_{i}}\;$Is the output of the third layer and $p_{i},r_{i},\;q_{i}$Is a set of linear equation parameters that can be adjusted in this layer. The parameters of this layer are called tally parameters.

**Layer 5**: The only node of this layer is a fixed node that calculates the main output of the network by collecting inputs to the node as Eq. (7).

(7) $$Q_{5,i}=\sum_{i}\bar{W_{i}}f_{i}=\frac{\sum_{i}W_{i}f_{i}}{\sum_{i}W_{i}}$$

**Fig 1:**
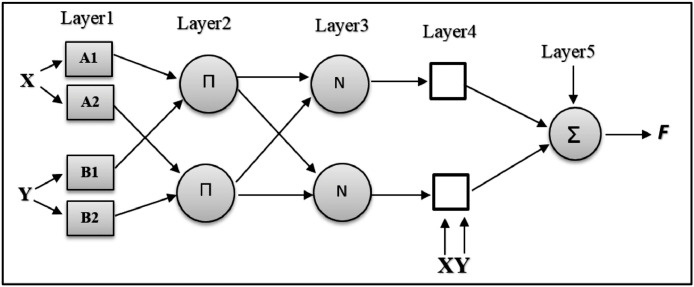
The structure of the Adaptive neural fuzzy inference system.

Also, the structure of this adaptive network is not unique and it is easy to merge layers 3 and 4 to form an equivalent layer and the structure consists of 4 layers. Fig. 1 shows the structure of an ANFIS used in this model.

In Fig. 1, each layer is specified in the order. The input values first enter the input layer. In this step, fuzzy membership functions are generated, then weights are formed in the second layer and applied to the output of the previous layer. In the third layer, relative weights are applied by fuzzy rules. In the fourth layer, network rules are applied. And then in the fifth layer, the final sum is presented in the form of network output.

### Differential evolution (DE) algorithm

**Fig 2:**
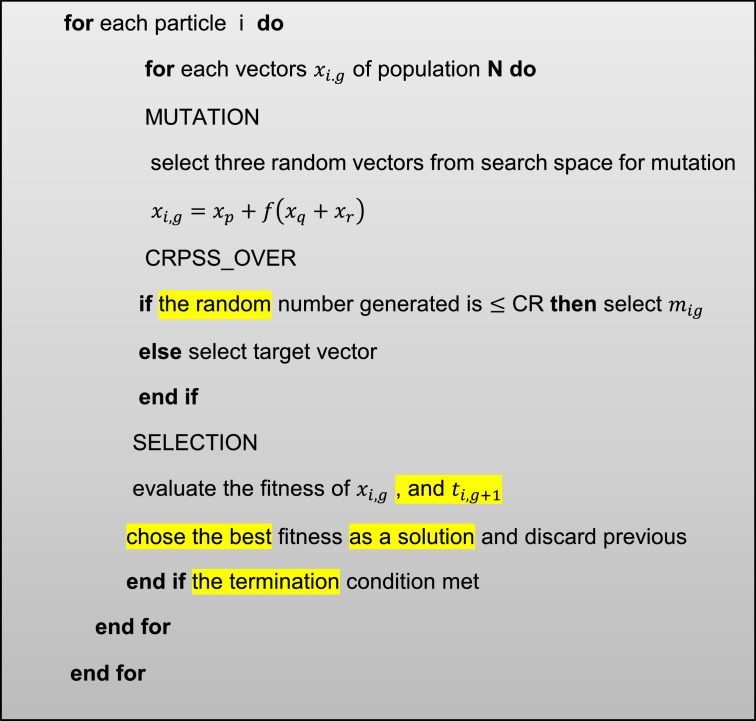
Pseudocode of the differential evolution algorithm.

The differential evolution of the population-based stochastic optimization algorithm was first proposed by Saturn and Price [15], which due to its high speed and good power in solving problems and simplicity, has many applications in solving optimization problems. Algorithm Unlike other algorithms, the mutation operator is first created to create a population of children and then the intersection operator applies to the members of the population [16] (Fig. 2).

### Mutation operator

In the differential evolution algorithm for each vector $x_{i}\left(t\right)$so that i={1,…,$\;n_{s}$} is defined as a test vector$\;u_{i}\left(t\right)$which is used in the intersection operator to create the child vector $x_{i}^{{}^{\prime }}\left(t\right)$To execute the mutation operator, perform the following steps:

**Step 1**: For the vector $x_{i}\left(t\right)$of the members of the current population, select the target vector $x_{i1}\left(t\right)$of the members of the current population, so that i ≠ i1.

**Step 2**: Randomly select the two vectors $x_{i2}\left(t\right)$and $x_{i3}\left(t\right)$from the members of the current population, so that i ≠ i1 ≠ i2 ≠ i3 and $i_{2},i_{3}∼U\left(1,\;n_{s}\right)$.

**Step 3:** The test vector $u_{i}\left(t\right)$is defined as Eq. (8):

(8) $$u_{i}\left(t\right)=x_{i_{1}}\left(t\right)+\beta \sum_{k=1}^{n_{\vartheta }}\left(x_{i_{2},k}\left(t\right)-x_{i_{3},k}\left(t\right)\right)$$

So that $x_{i_{2},k}\left(t\right)-x_{i_{3},k}\left(t\right)$, Represents the difference vector of k and β is a positive number that controls the magnitude of the changes applied to the target vector.

### Cross over operator

To create the child vector ${\;x\;}_{i}^{{}^{\prime }}\left(t\right)$, the discrete combination of the test vector $u_{i}\left(t\right)$and the parent vector $x_{i}\left(t\right)$is used according to the Eq. (9):

(9) $$x_{ij}^{{}^{\prime }}\left(t\right)=(\begin{array}{c} u_{ij}\left(t\right)\;if\;J\in ∁\\ x_{ij}\left(t\right)\;otherwise \end{array}$$

**Fig 3:**
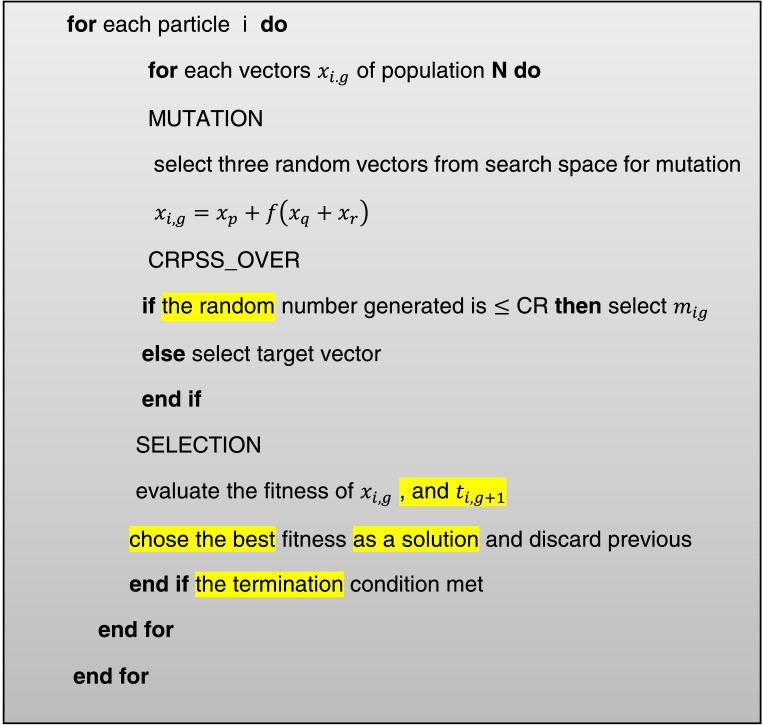
Pseudocode of the intersection operator algorithm.

So that $x_{\text{ij}}\left(t\right)$Represents the element j of the vector and $x_{i}\left(t\right),$∁ represents the set of Cross over points at which the Cross over operator is applied. In this study, the binomial Cross over operator is used. The intersection is Fig. 3.

If $n_{x}\;$represents the number of genes in the vector$\;x_{i}\left(t\right)$, the intersection is randomly selected from the set numbers $\left\{1,2,\;\ldots ,n_{x}\right\}$In the binomial method, $\rho_{r}$is the probability that each gene is selected from the test vectors. There is $u_{i}\left(t\right)$. The pseudo-code of the intersection operator algorithm is stated above.

Experimental studies show that DE/rand / 1 / bin, in which the target vector is randomly selected, creates good variation in the answers and has a good ability to converge the answers [17]. On the other hand, the DE/strategy current to best / 2 / bin will lead to convergence in the answers shown in the Eq. (10):

(10) ui(t)=xi(t)+β(x^(t)−xi(t))+β(xi2(t)−xi3(t))

In this relation, the first difference vector is obtained from the difference x^(t)of the best available vector with the parent vector $x_{i}\left(t\right)$and the second difference vector is obtained from the difference between the two vectors $x_{i_{2}}\left(t\right)$and $x_{i_{3}}\left(t\right)$Which are randomly selected are calculated [8]. Adjusting the parameters of the algorithm by trial and error and solving some sample problems $\rho_{r}=0.6,\;n_{s}=200,\;\beta =0.5$is selected which is a good performance in solving the proposed models [15–17]..

### ANFIS-DE

In this method, the differential evolution algorithm is used to determine the value of ANFIS membership functions that lead to the optimal training of this network. The advantage of this method is increasing the computational accuracy for the given topology. First, the data are divided into two data groups Training and test data are split. A significant percentage of training data is used to validate the model during training. Using sorted data, ANFIS training begins. The system training process allows this. Adjusts the parameters defined as the input or output of the model. The training process stops when the criteria set to stop the program are satisfied. After determining the training data, the type of membership functions, and the fuzzy inference system using The matching of the membership function parameters being optimized. In this paper, the differential evolution algorithm is used to determine the parameters related to the membership functions in the fuzzy inference system.

The number is then defined as the number in the membership functions (*N*) vector with *N* different dimensions. This vector contains the parameters of the membership function which are optimized by a differential evolution algorithm. The value of FITNESS FUNCTION defined in this study to The form is the MSE function. First, the parameters related to the DE algorithm are randomly determined, and then, using the algorithm, the specified values ​​are updated. At each step of the iteration, one of the parameters of the membership function is updated and these steps Continue for all parameters. After all, parameters are updated once, the next parameter is updated for the second time in the next iteration. Therefore, all parameters are updated repeatedly to reach the optimal point.

**Fig 4:**
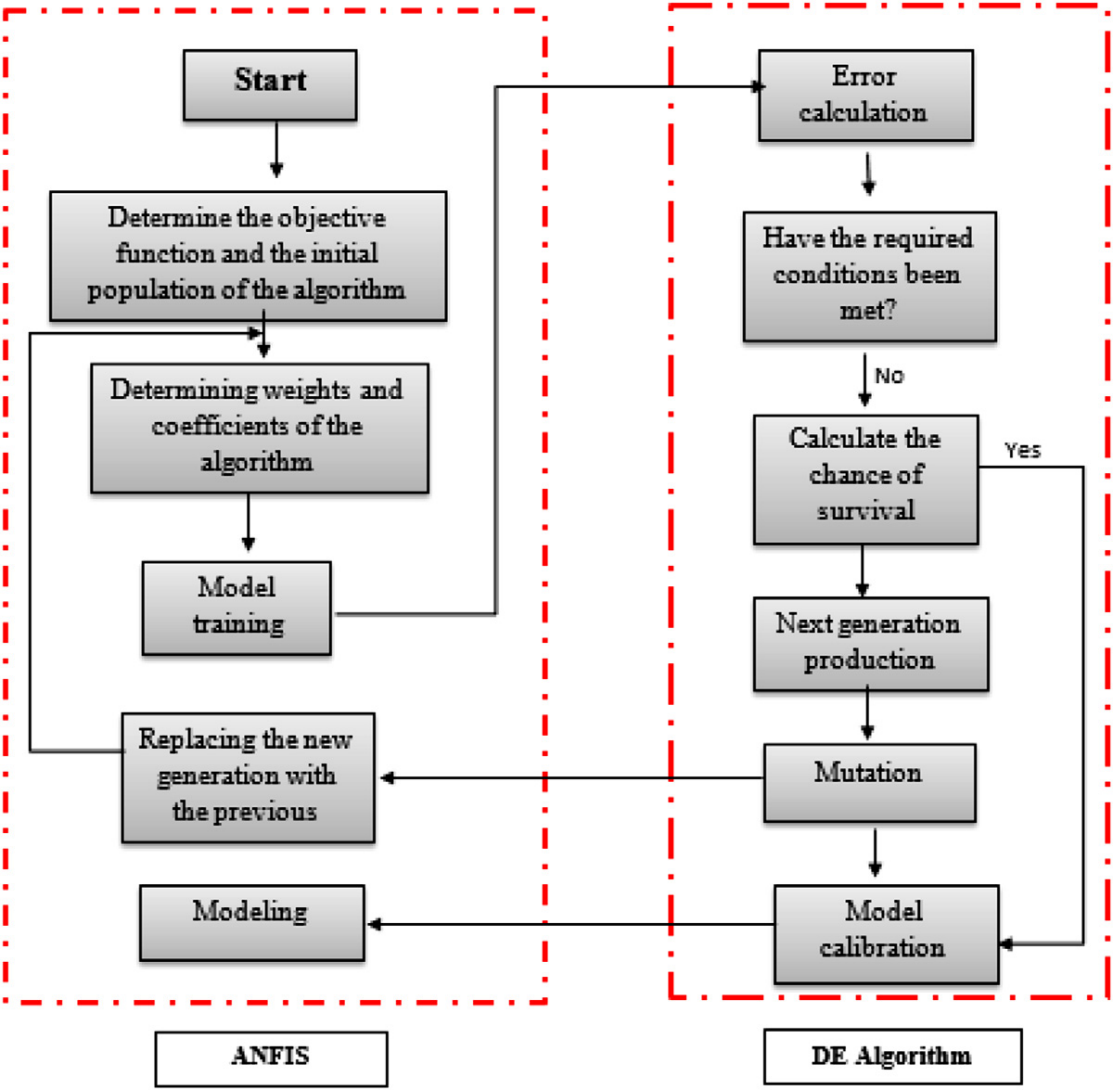
ANFIS network, flowchart optimized with the differential evolution algorithm.

Fig. 4 shows how to use the DE algorithm in the ANFIS network. This figure shows how to train the network by the DE algorithm.

In this study, according to the factors affecting the formation of inflation in the Iranian economy, the form of citizenship according to Eq. (11) was examined by the combined method of ANFIS-DE.

(11) y=f(x1,x2,x3,x4,x5,x6)

**Fig 5:**
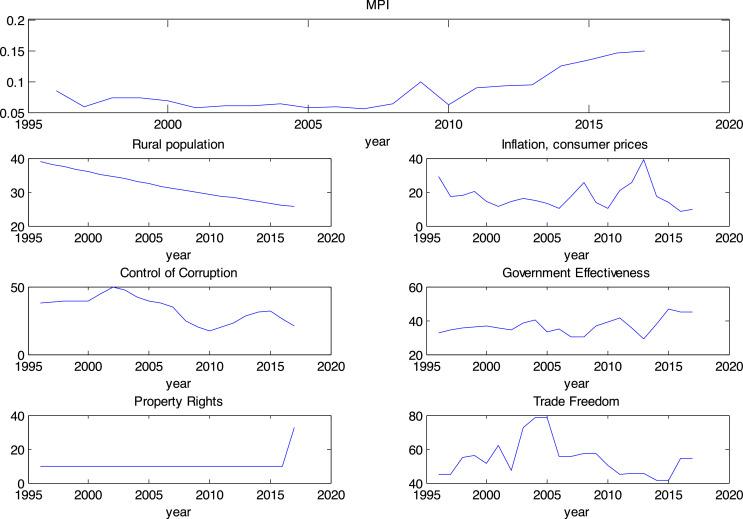
Charts for model variables.

Wherein Y: Multidimensional Poverty Index (MPI), x1: Rural population, x2: inflation, x3: control of corruption, x4: government effectiveness, x5: property rights, x6: Trade freedom. Multidimensional Poverty Indexes a dependent variable is presented in this paper. This data is annually measured by the United Nations for most countries in the world and is available on the UN site. The rural population ratio variable is one of the factors affecting poverty in Iran. These data are calculated and presented by WDI for Iran. In these data, the rural population is reported as a proportion of the country's total population. To study the effect of production on poverty in Iran, per capita GDP data were used. This data is obtained from WDI. Another factor affecting poverty is the general level of prices. Consumer inflation data were used for this purpose. This data is obtained from WDI. Data on corruption control and government effectiveness ratings were used to observe the effect of corruption and government on poverty in Iran. These data came from World Bank Good Governance indicators. Also, data related to the observance of property rights and the commercial liberalization index were used to extract MPI data in Iran. These data come from the Heritage Institute's economic freedom indexes (1996–2017). The values for the trend of these variables are shown in Fig. 5. Modeling of the Multidimensional Poverty Index in Iran was performed using the ANFIS-DE method by MATLAB software.

Fig. 5 shows the time series trend of each of the pattern variables in the period 1996-2018 for Iran.

**Criteria for evaluating results**

In this paper, advanced assessment has been selected as a criterion for studying poverty in Iran. As the complexity of poverty issues, the ANFIS method optimized with the DE algorithm for modeling has been selected. The data of this research area in the period 1996-2017. The designed network is based on 1996-2010 visual training data and is read using 2011–2017 data.

**Step 1:** In this step, all input and output variables of the model were normalized according to Eq. (12) [18,19].

(12) $$Z_{N}=\frac{Z_{R}-Z_{min}}{Z_{max}-Z_{min}}$$

Where $Z_{N}$Represents the normalized values of the variables and $Z_{R}$Represents the actual values of the variables. $Z_{\text{max}}$And $Z_{\text{min}}$Also, specify the minimum and maximum values of each variable in the time series. Table 1 shows the minimum and maximum values of each of the research variables.

**Table 1:** Normalized values.

	$Z_{\min}$	$Z_{\max}$
**Rural population (% of the total population)**	25.606	38.913
**Inflation, consumer prices (annual %)**	8.651258	39.26602
**Control of Corruption**	17.61905	50
**Government Effectiveness**	28.90995	46.63462
**Property Rights**	10	32.4
**Trade Freedom**	41.4	78.8

**Fig 6:**
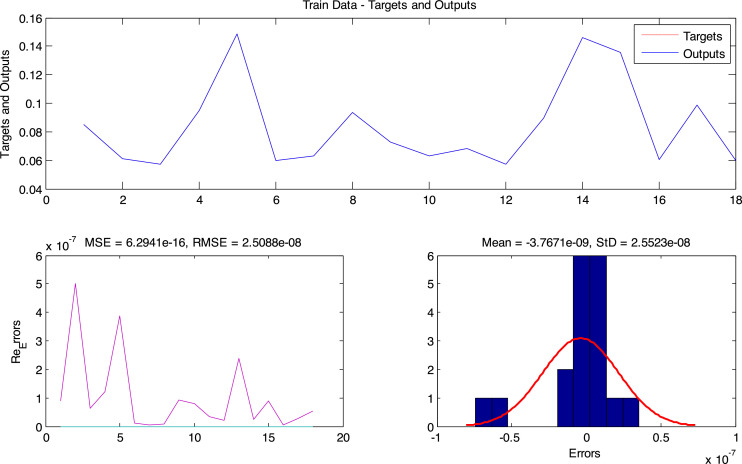
Modeling results on training data (1996–2010).

**Step 2**: According to the FITNESS function defined for the DE algorithm, the ANFIS_DE network was trained using the data 1996–2010. The network performance of this data is in Fig. 6.

Fig. 6 shows the performance of the ANFIS network against training data. In this figure, the output values and the target values are compared and the model error value is calculated for the training data.

**Fig 7:**
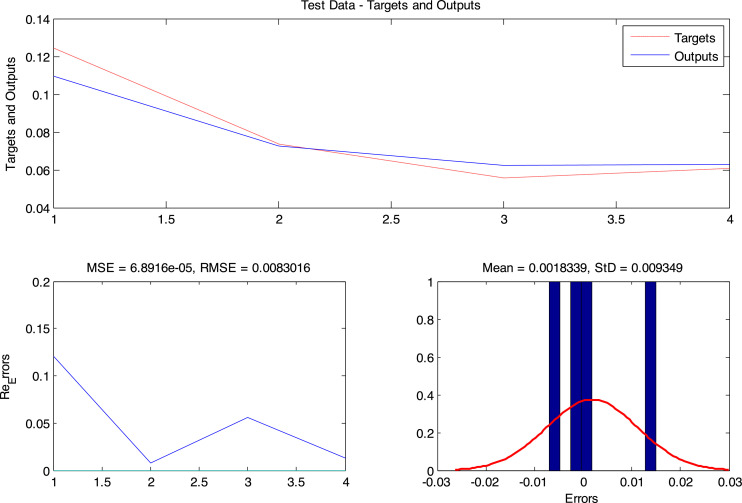
Modeling results on testing data (2011–2017).

**Step 3**: The trained network was then evaluated using the data of 211-2017. The performance of the network against test data is as shown in Fig. 7.

Fig. 6 shows the performance of the ANFIS network against testing data. In this figure, the output values and the target values are compared and the model error value is calculated for the testing data.

Besides, to evaluate the modeling power of the ANFIS_DE method, error criteria according to Eqs. (13)–(17) were used. The results of these criteria are in Table 2 and the relative error of the training data is in Table 3.

(13) $$MSE=\frac{1}{33}\sum_{i=1}^{33}{\left(Target_{i}-output_{i}\right)}^{2}\;\text{Mean}\;\text{Square}\;\text{Error}$$

(14) $$\text{RMSE}=\sqrt{\frac{1}{33}\sum_{i=1}^{33}{\left(Target_{i}-output_{i}\right)}^{2}\;}\;\text{Root}\;\text{Mean}\;\text{Square}\;\text{Error}$$

(15) $$\text{MAE}=\sqrt{\frac{1}{33}\sum_{i=1}^{33}\left|Target_{i}-output_{i}\right|\;}\;\text{Mean}\;\text{Absolute}\;\text{Error}$$

(16) $$R^{2}={\left(\frac{\sum^{\left(Target_{i}-\bar{Target}\right)}\;\times \;\left(output_{i}-\bar{output}\right)}{\sqrt{\sum^{{\left(Target_{i}-\bar{Target}\right)}^{2}}\times \sum^{{\left(output_{i}-\bar{output}\right)}^{2}}}}\right)}^{2}\text{Coefficient}\;\text{of}\;\text{Determination}$$

(17) $$STD\;Error=\sqrt{\frac{1}{33}\sum_{i=1}^{33}(Target_{i}-outpu{t_{i)}}^{2}}\;\text{Error}\;\text{standard}\;\text{deviation}$$

**Table 2:** Modeling performance criteria.

	Train data	Total data	Test data
MSE	6.29E-16	1.25E-05	6.89E-05
RMSE	2.5E-08	3.5E-03	8.3E-03
MAE	1.52E-08	1.11E-03	6.1E-03
R2	0.9999	0.9866	0.9949
Mean Error	-3.76E-09	3.33E-04	1.8E-03
STD_Error	2.55E-08	3.44E-03	9.34E-03

**Table 3:** Relative error values of test data.

	Target data	Output data	Error	Relative error (%)
2011	0.0893	0.0893	−3.55121E-08	−4E-07
2012	0.0935	0.0935	−1.24837E-09	−1.3E-08
2013	0.0946	0.0946	1.8067E-08	1.91E-07
2014	0.1242	0.10924	−0.014960108	−0.12045
2015	0.1354	0.1354	−1.31011E-08	−9.7E-08
2016	0.1459	0.1459	3.72115E-09	2.55E-08
2017	0.1487	0.1487	5.76661E-08	3.88E-07

## Results

The purpose of this article is to model poverty in the Islamic Republic of Iran. For this reason, to have an accurate model for poverty in Iran, a combination of fuzzy networks and artificial neural networks with evolutionary algorithms was used. The results showed that the use of evolutionary algorithms to train fuzzy neural networks leads to improving their performance and increasing the speed and accuracy of modeling. Therefore, it is recommended that researchers use these methods to improve the results of modeling and prediction in non-classical problems. In this study, a domestic and international approach to modeling the poverty model was presented. The results of this modeling showed that indicators of trade liberalization and respect for property rights are effective in poverty. As a result, it can be said that the fewer barriers to trade in a country, the more the country moves towards globalization. That country is expected to experience less poverty. Therefore, the government must put the policy of removing barriers to free trade and membership in the World Trade Organization on its agenda. Also, the comparative advantages of the country should be identified and efforts should be made to strengthen them. In this regard, it is necessary to create an appropriate infrastructure, one of which is respect for the property rights of individuals. According to the results obtained in this article, it can be said that inflation increases poverty. One of the effects of inflation on the Iranian economy is to reduce the level of welfare and create instability in the country. Governments must also work on the efficiency of social security and welfare expenditures to increase the efficiency and transparency of information to reduce poverty. Also, upgrading rural infrastructure can help reduce poverty in the country.

## Declaration of Competing Interest

The authors declare that they have no known competing financial interests or personal relationships that could have appeared to influence the work reported in this paper.
